# “We ensure that children and young people have a place to be and something meaningful to do”. Sustainable development in the Norwegian riding schools

**DOI:** 10.3389/fspor.2025.1690419

**Published:** 2026-01-12

**Authors:** Aage Radmann, Mathilde Kronborg, Anna Sätre, Petra Andersson, Gabriella Torell Palmquist, Susanna Hedenborg

**Affiliations:** 1 Department of Teacher Education and Outdoor Studies, Norwegian School of Sport Sciences, Oslo, Norway; 2 Department of Sport Science, Malmo University, Malmo, Sweden; 3 Department of Philosophy, Linguistics and Theory of Science, University of Gothenburg, Gothenburg, Sweden; 4 The Swedish Equestrian Centers of Excellence—Ridskolan Strömsholm & Flyinge AB and Department of Educational Studies, Karlstad University, Karlstad, Sweden

**Keywords:** sustainability, riding schools, institutional change, challenges, equestrian sports

## Abstract

Sport plays a significant role in promoting sustainable development at both local and global levels. However, many sports struggle to meet sustainability demands. To foster change and make sport more sustainable, it is essential to understand the factors that promote or hinder sustainability within specific activities. This study aims to explore how different stakeholders in Norwegian equestrian sports perceive and interpret sustainable development. The discussion is informed by the three dimensions of sustainability—social, ecological, and economic. The study is based on qualitative data collected through eighteen in—depth interviews with representatives from ten riding schools in Norway. Additionally, observations were conducted at three riding schools, and steering documents from the Norwegian Equestrian Federation were analyzed. Institutional theory provided the analytical framework for interpreting how established practices and norms influence sustainability efforts. Findings indicate that the social dimension of sustainable development is strongly emphasized in steering documents, interviews, and everyday practices at Norwegian riding schools. Elements of the economic and ecological dimensions are present but less prominent. Notably, ecological sustainability is not prioritized in the sector's agenda. The results suggest that the resilience of social structures and the pursuit of legitimacy within the equestrian sector create friction that hinders sustainability—related change. To advance sustainability in equestrian sports, it is necessary to address institutional change—examining how entrenched practices can be challenged and transformed over time.

## Introduction and aim

Sport has an important role to play in contributing to sustainable development in society at both a local and a global level. According to the UN, sport can be a major enabler of sustainable development, especially when it comes to issues such as tolerance, respect and increasing the empowerment of women and young people. In terms of ecological sustainability, the UN emphasizes that sport can contribute in a number of ways, for example: at events, by influencing sustainable food choices, reducing food waste for its members and spectators and by limiting the use of disposable items. Sport can also contribute to sustainable development through the shared use of facilities by different sporting activities and ensuring its facilities are energy efficient and, where possible, use renewable energy. Sport can also offer an effective educational platform for sharing information about water management in a climate-efficient manner (1). In contrast to the UN expectations research shows that the sports sector has difficulty meeting the demands of sustainable development (2). Sustainability goals are often in conflict either with each other or with the practical logistics of running sport organizations (3–5). In addition, sports activities have a number of specific challenges in meeting ecological goals. For example, many sport activities leave considerable ecological footprints as they are highly dependent on transport. The size of the footprint depends on where facilities are located and at which level the sport is played (6). Similarly, sport's relationship to social and economic sustainability varies. This can be due to participation costs as well as social expectations (such as gender constructions (7).

To bring about change and make sport more sustainable it is important to extend our knowledge of the promoters and challenges for specific activities. The aim of this article is to increase this knowledge with a specific focus on how stakeholders in Norwegian Equestrian sports understand and make sense of sustainable development. To date, research on Norwegian equestrian sports, and more specifically the Norwegian riding school, is scarce.

## Riding schools in Norway

In Norway, horse riding is one of the most popular sports and equestrian sports serve multiple functions, providing recreational activities, competitive sport, entertainment, and employment opportunities for individuals of all ages. In the last 50 years, the number of horses has increased rapidly, from 20,000 horses in the 1960s to 125,000 horses in 2012.

Simultaneously, the horse sector has changed from a sector in which horses were used as working animals for agriculture, forestry, and transport to a sector in which horses are used for recreation (8, 9).

The largest organization within the Norwegian equine sector is the NRYF, the Norwegian Equestrian Association, founded in 1915. NRYF is the eleventh largest federation in the Norwegian Sports Confederation (NSC) with 32,000 members, the majority of whom are girls and women (89.5%). NRYF's activities are organized in 309 sports clubs where members can own their own horses for recreation and competitive purposes or learn how to ride at a riding school (10). Yet, it is difficult to establish the exact number of riding schools in Norway as there are riding schools organized both inside and outside of NRYF. The exact number is, however, of little importance for this study as the activities offered by the riding schools do not seem to differ significantly. Similar to the Swedish riding schools [that have received more attention by research, see for example (11, 12)], Norwegian riding schools offer riding lessons and classes on horse handling to children, youth, and adults. The participants are mostly girls, and they ride for one or two hours a week.

## Theoretical framework

Transforming our current lifestyles and ways of living present deep, unsettling and fundamental challenges, yet this shift is essential for reaching the 17 Sustainable Development Goals (SDG) highlighted in the UN's Agenda 2030. In Agenda 2030, the SDGs integrate three dimensions of sustainable development: economic, social and ecological sustainability (13). The three dimensions are interconnected but represent core aspects. The economic dimension contains issues related to satisfying basic needs, equity, economic viability, and prosperity. The social dimension focuses on social well- being, equity, justice, and participation. The ecological dimension emphasis the biological system, protecting natural resources, minimizing pollution and maintaining biodiversity. The integration of these aspects is the foundation for sustainable development and in practical work trade-offs are common. In a conceptual paper, Purvis et al. (14) aim to identify the theoretical foundations of the model and conclude that there is no single point of origin. They also underline that even though the often-used circular diagram was first published in 1987, the conceptualization predates the publication. This is explained by the sustainability discourse arising from various schools of thought, which entails that sustainability is difficult to operationalize in research. This also applies to the more complex model of the UN SDGs. In our study we are interested in how the interviewees understand and make sense of sustainability in relation to economic, social and ecological sustainability.

In the concluding discussion we will discuss possible promotors and challenges to change towards sustainable development. Insights from institutional theory inform the discussion as institutional theory can be used to understand the resilience of social structures (15). By analyzing formal and informal norms, routines, and cognitive patterns, frictions towards change can be identified. Hence, the theory opens up understanding of why transformations do not occur, even though changes are urgent. Institutions are social structures with a high degree of resilience and must conform to certain rules and belief systems to receive legitimacy. This also includes non-governmental organizations and social organizations that, like corporations, “look to their peers” for cues to appropriate behavior rather than optimize decisions and practices. Scott (15) distinguishes between three institutional pillars:

1. The Regulative Pillar: Laws, regulations, and economic incentives (e.g., animal welfare legislation, environmental requirements for horse management).
2. The Normative Pillar: Professional standards and industry norms (e.g., expectations for animal welfare, sustainability practices in riding clubs).
3. The Cognitive Pillar: Cultural interpretations and collective understandings of horse management and sustainability (e.g., traditional vs. modern approaches to horse care).

## Previous research

In the following, studies on horse riding activities relating to the sustainable development dimensions are presented. We have organized the presentation of previous research in three sections connected to the sustainability dimensions (even though the concept of sustainability dimensions or the SDGs are not mentioned in the studies) to give the reader a picture of how these dimensions have been treated in presiouvs research.

In relation to the social dimension of sustainability, previous research shows that participating in a riding school and joining a stable club can enhance various skills in youth, including improved emotional regulation. The proximity to horses or other animals heightens awareness of feelings, necessitating the development of effective emotional regulation skills, as animals easily perceive and are influenced by human emotions (16, 17). Additionally, involvement in stable activities instills a sense of responsibility, as youth learn to care for the horses, fostering leadership and responsibility. Taking part in the activities requires emotional regulation, responsibility, and the ability to handle stress, presenting youth with multiple challenges during their time at the riding school (17, 18). According to the UN Convention of the rights of the child young people's voices should be taken into consideration. This is not always the case in riding schools. Research has shown that making one's voice heard is conditioned by social cultural factors and on how riding instructors conceptualize the young rider (19). Another challenge is the fixation on appearance. Having certain types of clothes can give status to the youth, negatively impacting the social aspect of the stable (11). However, successfully managing the challenges presented above contributes to the development of confidence and self-efficacy among youth (17, 18).

Research on economic sustainability shows that participation in sports and recreational activities over-all in Norway (and other countries) is conditioned by socioeconomic conditions (20, 21) and a specific child's parents' financial situation, especially if the child wants to participate in multiple sporting activities (3, 22). Equestrian sports are the most expensive sport activities for children and young people in Norway, averaging NOK 12,560 annually, followed by ice hockey at approximately NOK 11,000. The high costs are due to equipment, facility use, and competition expenses, and the economic barriers that can limit access to certain sports, raise concerns about social inclusion (23). Despite high costs for participants, economic sustainability is a challenge for the riding schools too. Many riding schools have economic challenges related to the high costs of the facilities and horses and the demand for employees taking care of the horses and the participants (24).

Research on ecological sustainability and horse riding is still scarce. Research has, however, shown that horses can contribute to sustainable development by promoting biodiversity (25). However, people's handling of horses can also contribute to a negative impact of local ecosystems through nutrient leakage from manure piles, pastures, and grazing fields (26, 27). Additionally, human activity within the sector influences climate emissions, particularly through transportation. Despite growing awareness of these sustainability challenges among stakeholders in equestrian sports, transformation remains difficult to achieve (28, 29).

## Method

To increase knowledge on how stakeholders in Norwegian Equestrian sports understand and make sense of sustainable development we have used qualitative research methods: semi-structured interviews with representatives from ten different riding schools, observations at three riding schools in Norway and text analysis of steering documents from Norwegian Equestrian Federation NRYF. The methods triangulation is combined with investigator triangulation (30–32). The authors of this article come from diverse academic backgrounds, including sociology, gender studies, education, psychology, philosophy, and sport science, and have contributed with various insights and ideas during the preparation, collection, and analysis of the source material. Furthermore, their experience of equestrian sports and the riding school context vary. Four have a long experience of equestrian sports and the riding school context, whereas two of the authors are new to the area. This has contributed to an increased understanding of what is specific to the sector in relation to other sporting activities.

### Steering documents from NRYF

The equine sector consists of various actors—from private horse owners to organizations like the Norwegian Equestrian Federation (NRYF) and agricultural authorities—operating within an institutional framework shaped by both formal regulations and informal norms. Here we focus on the steering document from the equestrian sport federation, NRYF.

Riding schools in this study are members of the NRYF and the steering documents used are produced by the federation. The documents are presented at NRYF's website.

There are documents related to laws and the regulation of the organization of a sport federation, steering documents for competitions, antidoping, marketing and children's sport (33). There is also a policy document including statements on how Norwegian equestrian sports should be developed in the period 2023–2027 (34).

### Interviews

The Norweigian website “rideskoler.no” (in English: Ridingschools.no) was used to find the riding schools. The website is a collaboration between NRYF, Bransjeforeningen for Rideskoler (The Industry Association of Riding Schools) and Norsk Hestesenter (The Norwegian Equine Center) and presents (among other things) information about location and addresses (online and offline) of riding schools in Norway. This information was used to map size and location of the riding schools as we wanted to find riding schools of different sizes for our study. To find the interviewees, we started by contacting 25 medium-sized (15–30 horses) and large-sized (more than 30 horses) riding schools in in East- and Southern Norway. Our choice of size was determined by our pre-understanding of that these riding schools were able to set aside time for answering our questions. Out of 25 contacted riding schools, ten responded positively and agreed to participate in the study. The sample is therefore, to a certain degree, self-selected. However, based on our experience in the equine sector we assessed the chosen riding schools to be representative of medium sized and large riding schools in Norway.

All interviewees were women and managers of the riding schools. Most of the interviews took place between August 2022 and December 2023, with three additional interviews conducted between February and April 2023. The average duration of each interview was approximately 45 min. The 18 interviews were carried out by one or two researchers from the team. The interviews were semistructured and we used an interview guide containing questions such as: Can you describe your riding school and the work you are doing here? How do you work with sustainable development? Where do you wish the equine sector to be in the next 10–50 years? At the end, the interviewees were asked if there was something they wished to add. The interviews provided the participants with the opportunity to articulate their perspectives on sustainability in the stable [see (35)]. Adhering to a methodological framework outlined by Berg (36), a checklist was employed to guide the interviews, ensuring that each identified theme was systematically addressed. Importantly, this approach provided a balance between structure and flexibility, permitting a dynamic and responsive discourse.

Interviews were either recorded via Zoom or conducted in person at the stable, based on the interviewees' preferences and logistical considerations. In-person interviews took place in quiet rooms with no other participants present, to guarantee confidentiality and anonymity. Subsequently, all interviews were transcribed.

### Observations

In the fall of 2023, participatory observations were conducted at three riding schools. The riding schools that had previously participated in the interview process were contacted via email to request permission for observations. Out of ten, three responded positively. Observations were carried out over five days at each riding school, with an average of five hours spent each day. This was done to understand the structure and weekly operations of the riding schools (37, 38). The observations allowed for a comparison between the interview data and the actual practices in the stables. The researcher actively participated in the daily activities at the stables. An observation guide was created to structure the data collection process. The researcher took notes and recorded the observations on a tape, which were transcribed later in the day to ensure accuracy and detail. Observations were conducted both during the day and in the evening to capture the full range of activities, as riding schools typically have different schedules and lessons throughout the day and evening.

### Analysis

We approached the source material (interview transcripts, observation notes and documents) abductively. Software for the analysis was not used. Instead we started with reading and re-reading the material and continued with a more focused thematic analysis to construct patterns and themes following Braun and Clarke's six-phase thematic analysis framework, which includes (1) familiarization with data, (2) generating initial codes, (3) searching for themes, (4) reviewing themes, (5) defining and naming themes, and (6) writing the report (39, 40). However, before phase six (writing the report) we used two more steps. First we introduced the sustainability dimensions to our analysis and tried to relate the source material to the core aspects (recognizing that they sometimes are interconntected). Issues related to satisfying basic needs, equity, economic viability, and prosperity, were seen as signifying the economic dimension of sustainability. When the interviewees talked about social well-being, equity, inclusion and participation, we interpreted this as being part of the social dimension. When we found traces of discussions connected to the biological system, protecting natural resources, minimizing pollution and maintaining biodiversity this was seen as belonging to the ecological dimension. Second, we organized the results in relation to Scott's (15) model of three pillars.

The process involved multiple rounds of review and discussion with the researchers in the study to ensure a thorough understanding of the source material. Thereafter the coded segments were organized into distinct themes. Codes that did not align with any themes or were infrequently mentioned were excluded from the analysis (39, 40). The themes underwent further refinement and definition, which included revisiting the data to ensure coherence and representativeness.The research team collaborated to finalize the set of themes, ensuring they captured the most meaningful patterns surrounding attitudes towards sustainability in the stable. This iterative process aimed to provide a comprehensive understanding of the data and facilitate the identification of key insights related to sustainability in Norwegian riding schools.

In the results section, we will analyse interviews, observations and steering documents integrated. We use quotes and research narratives to present our results. The research narratives, in this article, consist of fictional stories based on our field notes from the observations. The intention is that, by using a literary form, make the analysis of the observations more accessible to the readers. Fiction allows the results to engage the reader emotionally and through fiction we try to show the complexity of work in a riding school in relation to the the sustainability dimensions [see (41–43)].

### Ethics

The study was approved by Sikt (the Norwegian Agency for Shared Services in Education and Research) and adhere to relevant ethical guidelines (44). We have assessed that the processing of personal data will meet requirements in data protection legislation (45). Ethical considerations were carefully addressed throughout the research process to ensure compliance with ethical standards for conducting interviews and observational studies. Prior to the interviews and observations, all participating stables were provided with detailed information about the study's purpose, methods, and intended outcomes. The researchers obtained informed consent from all participants, emphasizing their right to withdraw from the study at any time without any consequences. To further protect the rights and dignity of participants, all data collected were anonymized, ensuring that no personal or identifying information could be linked back to specific individuals or stables. The researchers adhered to the principles of confidentiality and transparency, clearly communicating how the data would be stored, analyzed, and used in publications. Additionally, the observational methods were designed to minimize disruption to the participants' daily routines, respecting both their time and environments.

## Results

### Three overarching themes in steering documents, interviews and observations

Our analysis of steering docuemnts, interviews and observations indicates that NRYF and the riding schools present their activities related to three overarching themes. First, it is underlined that riding schools offer various activities for people of all ages. Not least the social aspects are underlined. The riding school is presented as *an inclusive social arena*. Children and youth are the main target groups; however, riding schools have activities for adults and elderly as well as for people with disabilities. Second, it is emphasized that the riding school is a place where people learn different things: the riding school is *an arena for learning*. Various activities are described, and the learning goals are related to concepts such as self-esteem, confidence and self- efficacy. Third, riding schools are presented as *arenas for promoting health and well-being*, where various activities are offered to various groups to promote their health and well-being.

#### The riding school as an inclusive social arena

In this section, the riding school as *an inclusive social arena* will be presented. This theme was identified as central across steering documents, interviews and observations.

The NRYF steering documents relate to laws and regulations and are focused on how sport federations should be organized, competitions, marketing, antidoping and children's sport. There are few guidelines regarding the operation of riding school activities, but it is underlined that a riding school should be inclusive, regardless of age, race, gender, or disability (34). In addition, they articulate core values revolving around respect, responsibility, safety, and knowledge, related to the horse, the organization, and other humans. They also emphasize the importance of demonstrating horse welfare in all aspects of their activities (34). Furthermore, development of low threshold activities, activities that will attract people with a foreign background are mentioned together with activities that attract people throughout the lifespan. It is also underlined that all members should have access to good facilities. “Good” is related to the facilities' adaptation to the offered activities. The importance of increased public funding of the facilities is emphasized, increased knowledge in relation to maintenance of facilities and the use of multipurpose facilities are emphasized.

A constructed narrative based on field notes from the observations illustrate the riding as an inclusive arena too.

The people were very welcoming when we arrived at the riding school right outside Oslo. We were met by children and young people—mostly girls—as well as parents who laughed and talked to each other while brushing the horses and preparing them for the riding lesson. It seemed that the riding school was permeated by including attitudes. We discussed with each other how they had been successful in creating this environment. In other stables, we had seen a set of rules—known in that stable as the 10 commandments—or notes on how to behave. These were primarily focused on social conduct such as being kind to one another, helping each other, and keeping the stable tidy. These rules were painted on the walls and hung around the stable as reminders. Including and positive attitudes were also found between the employees, and we were told that they always started with complementing each other for something they had done. Maybe all riding schools we visited had worked with these attitudes, we asked ourselves. (Narrative based on fieldnotes from observations at riding school 1, 2 and 3)

The interviewees told us repeatedly that they worked to make the riding school as accessible as possible for all, regardless of age, economic opportunities and disability. According to them, an overarching goal for the riding schools was to provide a safe and inclusive space where children and youth can spend time, develop skills, and build social connections. Their stories centered around the riding school offering a variety of structured activities to make the stable a recreational hub for young people, including evening riding lessons and summer camps. The stable was presented as a meeting place—a social arena where children and youth can engage with horses and each other in a structured but relaxed setting. The importance of this arena was emphasized by those working in the stables:

I'm passionate about riding and instruction, but also about what this place represents for so many children. It's a meeting place. Right now, we have around 60–70 girls who care for our horses and use the stable as a kind of youth club. I see that as an important mission—ensuring that children and young people have a place to be and something meaningful to do. (Interview 11)

The quote highlights how the interviewees underlined the importance of the stable environment for young people—rather than what happens during the riding lesson—not least for young people's sense of belonging and their social well-being. There are riding schools that serve as social arenas for kindergartens, schools, and elderly homes. Some riding schools deliver classes for schools to learn how a stable is run, whereas others organize visits for young children or older adults so that they can spend time with animals. Some riding schools actively integrate elderly individuals through structured activities such as dedicated riding lessons, carriage rides, and social gatherings.

The importance of working towards the stable being a social arena for all is also underlined in relation to children and young people's various economic opportunities. As we have already mentioned, horse riding at a riding school is one of the most expensive sport activities for children and young people. The interviewees are aware of this but describe how they try to give all children and young people opportunities to take part of the social arena.

We offer a wide range of activities for children and youth in the village—not only riding lessons but also by providing a welcoming space where they can simply be. Many love horses but don't have the opportunity to own one, and this gives them a chance to be involved. (Interview 8)

Another interviewee reflected on her commitment to broadening access to equestrian activities, ensuring that engagement is not limited to those with familial ties to the sport in this way:

Equestrian sport has long been a space for people who might otherwise feel excluded. While structured, strategic efforts to enhance inclusion have increased in recent years—particularly through green care initiatives and partnerships between stables, local municipalities, and county authorities—this setting has always offered structure and support for those in need. (Interview 14)

The “green care initiatives”, in the quote above, refers to the green sector (agriculture) and the health-promoting activities in the “Green care farms” that are part of the Norwegian welfare system. The connection between nature and well-being is central to the concept of *Green Care*—the structured use of nature-based interventions to promote physical, mental, and social health (46). The green care farms (in Norwegian, “Inn på tunet”) are required to comply with specific health, environment, and safety regulations (47). The activities are offered to, and adapted to all levels of knowledge and ages. Schools, families, psychological health and people with cognitive challenges are identified as important targets for the activities. The activities are aimed at upbringing and education, work and work training as well as health and care (48). The interviewee quoted above also mentions that collaborations between different partners have facilitated the opening of the stable activities for various groups. She points out that the activities at the riding school do not only include children and young people.

While some stables focus primarily on youth, others emphasize the importance of providing opportunities for older adults to engage in equestrian activities. The interviewees highlight the potential of riding schools as intergenerational meeting places, promoting physical activity, mental well-being, and social inclusion for elderly adults alongside younger participants.

We have a program for elderly individuals where they visit the stable, interact with the horses, take a carriage ride, and then come inside for coffee and waffles. (Interview 9)

Furthermore, the interviewees talked about how some groups, in the quote below people with disabilities, was offered jobs. This was possible as the riding school collaborated with other actors.

We have projects aimed at helping people reintegrate into society by giving them a place to go and meaningful tasks to do. This is not about riding lessons, but rather about taking care of the horses and stable work. Through one initiative, we have hired five individuals with mild disabilities as part of a government-supported employment program. (Interviews 9).

According to the interviewees the riding school—its sensory environment, daily routines, and relational dynamics—offers a unique setting contributing to inclusion of all.

#### An arena for learning

Except for the social aspects of the activities, learning aspects were stressed in interviews and in the practice that we observed. In this section we will present how the riding school is presented as *a learning arena*. In the narrative below one of the parents that we met during an observation talk about the riding school as a place where children learn for life.

During one of our visits to the riding school a group of very young children were in the stable. We were caught in conversations with the teachers and an accompanying parent about what the children were to learn in the stable. One of their teachers told us they brought the children to the stable every week so that they could be physically active and learn how to interact with animals. Another teacher said that they also brough older children to the green area for informal learning. One of the parents told us that she thought it was good that the children were able to do the preparation work, as this was seen as a way to develop leadership skills and gain confidence.

These skills were something that the children could bring to other arenas of their lives. (Narrative based on fieldnotes from Riding schools 1, 2 and 3)

The interviewees underlined that from an early age, children are given significant trust and autonomy in completing their stable-related tasks. They emphasized the importance of making young people feel responsible and building their confidence. In addition, interviewees underlined how working with horses fosters a sense of mastery and psychological well-being.

It gives such a sense of mastery—being able to handle a large animal independently. It builds self-confidence to know that you can manage it. (Interview 9)

They point out the positive challenges young people meet in the stable environment and just like the parents we met in our observations they underlined that it was important for the youngsters to try to do different activities, even if they did not succeed in them. Even in the face of setbacks, such as falls or challenges in learning, the goal is to make the stable a place where children feel encouraged to return and continue their engagement with horses, she/they said.

I'm happy to come here. I always want to come back. Yes, sometimes you fall off, get hurt, and cry a little. But the point is that it should still be fun to come back. (Interview 4)

Riding schools also provide alternative education for children and young people struggling in traditional school settings. Through hands-on learning in the stable, these children develop practical skills, social competence, and self-confidence according to the interviewees.

We have several programs related to mental health that allow children to participate in stable activities and experience a sense of contribution. One of our key initiatives is the 'school project,' in which schools identify students who struggle with the standard curriculum—whether due to attention issues, bullying, or feelings of exclusion.

Groups of 4–6 children come here one day a week, where they engage in learning through stable work. We align activities with the school curriculum, so the teacher accompanies them, and they complete preparatory and follow-up tasks in school. For example, in one lesson, they learned about chickens by gathering eggs and making an omelet. They even incubated eggs in the classroom. Once these school children feel comfortable and understand the routines, we invite entire school classes. The small group then acts as assistant teachers, which shifts the dynamics at school. Those who were previously seen as outsiders suddenly gain status and confidence, positively impacting their school experience. (Interview 9)

It was pointed out that the riding school was a place where children and young people learn skills that can be used in other areas. In the interviews, the stable environment, with its routines and responsibilities, was described as strengthening both self-esteem and self- efficacy. The participants emphasized that the activities give the participants a sense of accomplishment and purpose, particularly when contributing to the horse's care—an experience especially meaningful for those facing loneliness or mental health challenges.

#### The riding school as an arena for promoting health and well-being

In this section we will present how our observations and interviewees point to the riding school as an arena where health and well-being are achieved.

During the afternoon several different activities were arranged in the riding school. At one time an extra person, a physiotherapist, worked together with the riding school instructor to create a learning environment for children with disabilities. The physiotherapist told us that she tailored the lessons to everyone's skill level. The parents we met in the riding school expressed gratitude for this and one of the mothers said that her daughter's confidence had increased since starting the program. Like all children and young people, these young people were expected to prepare their own horses by brushing and saddling them. We were also told that in the evening, a girl with special needs assisted the stable guard a few days a week. (Narrative based on field notes from riding school 1, 2 and 3)

That riding schools offered activities tailored to promote health and well-being for individuals with physical and psychological disabilities was not only seen in the observations. According to the interviewees the riding school can be seen as an arena for well-being across all age groups.

It is important to care for children and young people, but it is equally important to support adults. Many adults also need a place to meet others, engage in meaningful activities, and feel a sense of belonging. These riding courses offer a social experience, but also provide physical, mental, and emotional benefits. (Interview 11)

That riding schools arrange activities related to the health sector is well known and Equine Assisted Activities are offered in many riding schools around Norway. Some of the activities are economically supported by public health and some of them are paid for by the participants. There are many kinds of activities, such as riding lessons for children with different physical and mental disorders and one of our interviewees emphasized the therapeutic value of horseback riding.

As a physiotherapist, I have studied the body extensively, and I have also been involved in other sports. The way the body interacts with the horse is fascinating. Riding is one of the fastest ways to train the spine, core, and back without conscious effort. Therapeutic riding is particularly beneficial because it requires minimal verbal instruction—what matters is ensuring that the rider sits correctly and follows the horse's movements. Over time, they naturally develop better posture and coordination. And of course, there is great joy in it. (Interview 6)

The interviewees present strong beliefs in the therapeutic value of horses, also for mental health. Horse riding is only one of several activities arranged to promote health and well- being, just being with the horse is seen as beneficial.

We joke a little in the stable that we don't need a psychologist because we have the horse. And honestly, it feels true. (Interview 16)

There are also activities for adults who are unemployed due to different circumstances who get a chance to care for the horse, walk with the horse, carriage ride, or just participate in the daily activity of the stable. While horses have long been used in physiotherapy, there is growing recognition of their value in mental health care. The shift reflects an evolving understanding of the stable as a therapeutic space—one that supports not only physical rehabilitation but also emotional resilience and social connection. According to one of our interviewees this is a welcome development.

If we look 10 years ahead, I hope it will be more common to use horses in therapy— not just for physiotherapy but for mental health as well. (Interview 17)

The stable environment is also used for psychiatric day care programs, offering a calm and structured setting for individuals in need of support. One of the interviewees said that:

There are people we see regularly, whom we greet, but who mainly come here because of the peaceful atmosphere. There is a psychiatric day center nearby, and they use the stable area for walks and relaxation. (Interview 2)

Other activities described by the interviewees are work placements for individuals who have faced social exclusion or require government assistance to re-enter the workforce. According to the interviewees, these programs have been well received and recognized for their social impact. In the following quote the riding school manager explains that to start with the riding school employed this group because they were cheaper to hire than others, but later on the importance of how beneficial the contact with horses can be has overshadowed the economic reasons.

Yes, I suppose this has always been part of the riding school environment—many people have been given opportunities for work training here. Initially, this may have been due to economic factors, as hiring part-time staff was necessary. However, there is something special about horses. People have long recognized their impact, but now there is growing awareness of how beneficial they are for individuals. Many have found their identity and purpose through working in the stable. When I ran my own riding center years ago, I even had individuals from the prison system working there. (Interview 5)

In addition to the psychological benefits, the stable was described as a place of peace and restoration within the urban landscape. One of the interviewees used the concept “green lung” to describe this

It's a small green lung. We have a lot of trees, bushes, and open space here. It's a recreational area—a large one, right in the middle of the city. (Interview 16)

The green lung concept refers to an area of parkland within a town or city, considered in terms of the healthier environment it provides. However, the interviewee seems to connect it to “green care” (mentioned above) rather than a place where plants release oxygen through the process of photosynthesis or a place in which a balance of oxygen and carbon dioxide can be created in the atmosphere.

## Concluding discussion

The aim of this article was to increase knowledge on how stakeholders in Norwegian equestrian sports understand and make sense of sustainability. We have identified three overarching themes in our analysis of the steering documents, interviews and observations: the ridings school as an inclusive social arena, the riding school as a learning arena and the riding school as an arena for promoting health and wellbeing. It is now time to elaborate on how these themes relate to the three dimensions of sustainability. A first conclusion is that all three themes are connected to the social dimension of sustainability. Social well-being, equity and inclusion were strongly emphasized in the source material over all. When the riding school is presented as an inclusive arena, working with including different groups, lowering thresholds and building arenas for all are referred to. In the second theme, the riding school as a learning arena, learning related to how people should act towards each other and how they can become good (inclusive) leaders (rather than how they can become future Olympic medalists) is underlined. The promotion of health and wellbeing (theme three) is also filled with ideas on how to organize activities for all. There are traces of the other sustainability dimensions too. In the steering documents economic sustainability is referred to in relation to lowering thresholds for people who cannot afford to participate, the possibility of public support to the facilities and elite sport. In addition, economic viability is important for the riding schools, yet this dimension does not seem to overshadow the social dimension. Satisfying basic needs and prosperity are not found.

The ecological dimension is not high on the agenda in either source material—this dimensions seems to be rather unreflected. The interviewees answer our direct questions about their work with ecological sustainability that they realize that they don't think much about this dimension. Even so, when asked they refer to the importance of preserving green areas for urban people. However, they do neither problematize the ecological footprint of the riding school activities nor their visitors' negative impact on the environment through transportation. Furthermore, the possible contribution of horses' feeding habits as contributing to biodiversity is not mentioned.

Finally we will turn to institutional theory to understand the riding schools relation to sustainable development. Summarily we can conclude that the social dimension of sustainable development is highly represented in all pillars (the regulative pillar as well as in the normative pillar and cognitive pillar), and that the ecological dimension is almost invisible.

The regulative pillar (15) of the Norwegian riding schools, i.e., steering documents from NRYF and the written notes presented in one of the narratives, underlines the social dimension. NRYF emphasises that equestrian sport is open to all and that activities should be adapted so that anyone can participate independently of age, social class or body ability. On a lower level, but still part of the regulative pillar are the ten commandments on how to behave found in several stables. These emphasise being kind and helping each other. It is also clear that the cognitive pillar is based on cultural beliefs that recognize the value of equine activities for all.

When the riding school practices (the normative pillar) are studied the social dimension stands out too. Riding schools provide structured activities for children and youth, fostering social connections and personal development. They create supportive environments for young individuals, but also for other groups of all ages. The economic dimension of sustainable development relates to inclusion. Costs of elite sport and competitive activities are not mentioned. Instead, economic sustainability is related to how stable activities can welcome groups who can't afford riding. Riding schools have difficulties to make ends meet. When they offer multiple different activities, this may be a way to increase the number of participants. They also underline the importance of collaborating with other stakeholders to achieve economic sustainability for the riding school.

The importance of the social dimension of sport is seen in other studies of Norwegian sports too [cf, (3)]. According to a study by Anne Tjønndal et al. (3), there are three overarching tensions within Norwegian sports and outdoor activities, the ecological and economic, social and ecological sustainability in practice, and social sustainability and the competitive focus of sports. In our study we do not find those tensions, possibly as the content of the three pillars are alined.

It is, however, interesting to note that even though our analysis point to that the riding schools are seen as an inclusive social arena for all, participation is to some extent conditional. As we mention in the beginning of the article, most riders in contemporary Norway are women and girls and NRYF underlines in their documents that sport shall be gender equal. However, NRYF does not present a specific goal in relation to gender equality and interviewees do not talk about inclusion in relation to gender and they do not mention specific activities organized for boys and men. In the steering documents activities related to recruiting members from other groups, such as people with a foreign background, are mentioned. Research from other Western European countries has pointed out that horse riding is a white activity and that children and young people (girls) with foreign backgrounds do not take part in these activities (49). We do not know whether special activities are arranged for these at the riding schools, but such activities are not referred to in the interviews. Nor do we see such activities in our observations.

It is also worth mentioning that even though the interviewees underline that they strive for children and young people to be included, the inclusion demands commitment. The interviewees say that in the riding schools there is a youth group called the “stable gang”. This group is presented as young people who have a strong interest in horses and their activities focus on teaching children how to care for horses and participating in daily stable routines. This is an important group for the riding school. However, these activities require that the young people involved spend extra time in the stable.

Perhaps our most important goal is to engage children and young people who have a strong interest in horses—those who spend extra time at the stable beyond their regular riding lessons. We call them our stable gang. (Interview 2)

It becomes clear that the more engaged you are with the horse, the more support and encouragement you receive to pursue that engagement—those considered the most interested and knowledgeable are the ones most included. That inclusion is conditional is also demonstrated in by Waerner et al. in a study on how Swedish horse-riding instructors construct youth in the riding school. Young people are given voice when they are seen as competent and interested (50). Sport, equestrian sports included, need to contribute to sustainable development and U.N. has even stated that sport can be a major enabler to this. For sport to take this role, change is essential. NRYF and stakeholders in Norwegian riding schools refer to norms, practices and ideas that can be related to SDGs 3, 4 and 10, but they are a long way from other goals. It is, however, important to recognize that the results are more complex and have to be related to how stakeholders make sense of their practices rather than just concluding that they do not live up to the SDGs as formulated by U.N and others.

Social sustainability is not only a relevant concern *within* the equine sector, but also an area where the sector can play an active role in promoting broader societal goals. By fostering inclusion, supporting mental and physical health, and strengthening interpersonal relationships, the equine sector demonstrates its potential to contribute meaningfully to social cohesion and resilience in society at large. But, despite the positive impacts of riding schools on social sustainability, challenges remain in achieving ecological and economic sustainability. The resilience of social structures and the need for legitimacy within the equine sector can create friction towards change. To understand how sustainability initiatives can be implemented in the equine sector, it is necessary to examine institutional change—how established practices are challenged and transformed over time. In this context, institutional change can be understood through four distinct phases as outlined by Greenwood et al. (51). The first phase, *awareness*, involves the emergence of criticism directed at existing practices. For example, in our case, this could include discussions about the environmental impact of equestrian sports highlighting the need for change. The second phase, *development of alternatives*, focuses on the introduction of new and sustainable models. In relation to the equine sector, this phase may include initiatives such as research projects exploring economically sustainable and environmentally friendly stables. The third phase, *mobilization of support*, occurs when influential actors begin to endorse the proposed changes. For instance, equestrian federations may implement and advocate for public economic support and ecological standards within the sector. Finally, the fourth phase, *institutionalization,* marks the point where the changes are adopted as standard practices and become widely accepted within the equine sector. By leveraging the phases of institutional change, stakeholders can work towards sustainable practices that are widely accepted and integrated into the sector. Future research should focus on exploring sustainable models and mobilizing support from influential actors to achieve institutionalization of sustainable practices within the equine sector.

## Data Availability

The data presented in this study are available on request from the corresponding author. The documents used can be found using our references, and transcriptions from the interviews are saved at the Malmo University server and not published OA because of ethical reasons related to protecting the anonymity of our interviewees.
